# Detecting Suicidal Ideation in Social Media: An Ensemble Method Based on Feature Fusion

**DOI:** 10.3390/ijerph19138197

**Published:** 2022-07-05

**Authors:** Jingfang Liu, Mengshi Shi, Huihong Jiang

**Affiliations:** School of Management, Shanghai University, Shanghai 201800, China; jingfangliu@shu.edu.cn (J.L.); 19720600@shu.edu.cn (M.S.)

**Keywords:** suicidal ideation detection, social media, ensemble method, multi-feature fusion, machine learning, Weibo, China

## Abstract

Suicide has become a serious problem, and how to prevent suicide has become a very important research topic. Social media provides an ideal platform for monitoring suicidal ideation. This paper presents an integrated model for multidimensional information fusion. By integrating the best classification models determined by single and multiple features, different feature information is combined to better identify suicidal posts in online social media. This approach was assessed with a dataset formed from 40,222 posts annotated by Weibo. By integrating the best classification model of single features and multidimensional features, the proposed model ((BSC + RFS)-fs, WEC-fs) achieved 80.61% accuracy and a 79.20% F1-score. Other representative text information representation methods and demographic factors related to suicide may also be important predictors of suicide, which were not considered in this study. To the best of our knowledge, this is the good try that feature combination and ensemble algorithms have been fused to detect user-generated content with suicidal ideation. The findings suggest that feature combinations do not always work well, and that an appropriate combination strategy can make classification models work better. There are differences in the information contained in different functional carriers, and a targeted choice classification model may improve the detection rate of suicidal ideation.

## 1. Introduction

Suicide is harmful behavior with self-directed death [1]. Despite the huge progress in modern medicine in diagnosing and treating major mental disorders, suicide remains a difficult public health problem [2]. According to the latest report of the World Health Organization (WHO), The Global Status of Suicide 2019, suicide is one of the principal causes of death among immature persons this day, with serious social implications. Abdulsalam et al. categorized suicidal behavior into suicidal ideation, suicide scheme, and suicide attempts [3]. Suicidal ideation is the initial unattempted plan, a suicide scheme is a technical method with a clear purpose, and a suicide attempt is an attempted behavior that can lead to death, all three of which are aimed at suicide and have increasing levels of depth. Suicide prevention requires rapid identification and intervention, especially during the COVID-19 pandemic when unusual lifestyles are affecting people’s moods, and many people around the world are suffering from severe depressive disorders and psychological distress, and they are more likely to develop suicidal ideation [4,5,6].

However, there is still a lack of effective methods to identify potential people with suicidal ideation as early as possible so that timely interventions can be made to prevent them from resorting to suicidal behavior [7]. In recent years, researchers have examined people’s mental health problems from two main perspectives [5]. One is based on a traditional perspective that relies on clinical interactions between medical staff and patients, using traditional scales and questionnaires to assess suicidal ideation. However, the drawback of this approach is that people are often shy or reluctant to consult a psychologist or counselor, suffer from deliberate concealment and misreporting, and do not disclose their plans before committing suicide [5,7]. Comparatively, suicide screening techniques through accessing and analyzing social media data is a growing and emerging field [8,9,10]. Previous studies have shown that, with the widespread use of the Internet, young people with suicidal ideation may disclose suicidal thoughts or seek information for support on social media [11,12,13]. With the recent popularity of social media such as Facebook and Twitter, there is a growing trend for young people with suicidal ideation to leave suicidal notes on social media [14,15]. Although it is unclear to what extent this online expression is comparable to physician-derived suicide risk, several studies have shown that online expression of suicidal thoughts is associated with psychologically assessed suicide risk [16,17].

Detecting suicidal ideation through social media may help public health professionals or psychologists quickly identify users with suicidal thoughts and intervene promptly. This idea has already been applied to a real dataset and received good feedback from psychiatrists [18]. For example, Chiang et al. developed an early warning system for detecting suicidal ideation based on social networking sites such as Facebook to identify users with potential suicidal ideation, thus helping psychologists to be able to intervene promptly [19]. Furthermore, the results of a case study suggest that clinical health professionals and psychologists are equally concerned about the new changes brought about by the emergence of social networking sites such as Facebook, and they identify users with suicidal thoughts based on the textual content they post and intervene immediately [20].

Here, we critically review previous research in which we developed a suicidal ideation recognition model based on a machine learning approach and applied it to an objectively existing microblogging dataset to identify suicidal posts based on online social media data in order to capture users who need intervention. Unlike previous studies in the literature, the model is based on a novel multi-feature fusion integration approach that examines critical predictors of the content posted by suicidal users. In addition, to comprehensively consider important information about suicide in posts, we used multiple methods to construct feature vectors, including basic statistical features of posts and suicide risk factors. In addition, we constructed new feature vectors by embedding clustering words into keywords. The original features were combined in different ways, and the best classification model was determined. Because of the differences and interactions between the feature function vectors, we designed variants of the stacked integration model to improve the recognition rate of suicidal ideation by fusing different functional modules.

## 2. Literature Review

Detecting suicidal ideation through social media is challenging [5]; therefore, we reviewed studies on suicidal ideation detection in social media and documented their methods, limitations, and model performance in Table 1.

### 2.1. An Overview of Methods to Detecting Suicidal Ideation

From a research perspective, the current methods for detecting suicidal ideation are mainly questionnaire-based and machine-learning methods. Stephanie et al. concluded that assessment questionnaires and scale-based models for predicting suicidal thoughts and behaviors (STB) are heterogeneous and generally effective. However, owing to the rise of social media this day, persons do post “suicidal” messages on social media platforms such as Twitter, which provides more objective data [17]. At the same time, the widespread use of new techniques in machine learning and natural language processing has made it possible to extract semantic information from text and speech, and this advancement provides potential predictors for STB prediction from the perspective of linguistic features [1].

In terms of classification method adoption, the simplest form of classification method is to divide some data instances into two categories using selected features. Most existing studies have used single classification methods to identify suicidal ideation, such as support vector machines [21,22,23], plain Bayes [18], logistic regression [24], and deep learning [16]. In recent years, integrated learning methods have also received close attention from researchers [27] and have been successfully applied to solve many problems [28,29,30]. Effective ensemble learning usually performs better than individual models [25]. In addition, ensemble methods can eliminate overfitting and improve the model’s overall performance. For detecting suicidal ideation in social media, few studies have attempted to apply ensemble methods to address this problem, and most ensemble methods do not adequately take into account the differences in the information contained in different underlying classifiers.

### 2.2. An Overview of Features to Detecting Suicidal Ideation

Detecting suicidal ideation using social media posts requires a thorough understanding of the key predictors of the content posted by suicidal individuals. Aladag et al. [26] tagged suicidal and non-suicidal posts in a public dataset on Reddit and extracted features using term frequency–anti-document frequency, linguistic queries and word count, and sentiment analysis of post titles and body text. In a study by Desmet and Hoste et al. [21], word packets, polarity dictionaries, LSA topic models, and named entities were selected as input features for the prediction model, and the final prediction results were comparable to those of manual annotation. In a study on suicide on Weibo, Cheng [22] mainly used Simplified Chinese Language Query and Word Count (SC-LIWC) to count the number of occurrences of each type of word in users’ posts, and investigated the association between SC-LIWC features and five suicide risk factors through logistic regression.

In existing studies, few studies have examined the way features are combined, although helpful information has been drawn from multiple sources. Different functional features can construct new feature vectors, and the new feature combination vectors may present different effects in different classifiers. A freezing technique was proposed in the study of Nguyen and Nguyen [31], where the feature vectors CNN-F and LSTM-F were generated by CNN and LSTM models, respectively. Experimental results show that the feature combination method has higher recognition accuracy than CNN-F and LSTM-F. By combining the existing features to form new features, it has attracted a lot of attention from researchers.

### 2.3. Critical Review

In summary, detecting suicidal ideation based on social media is an emerging research trend, and current research focuses on feature construction of text content and innovation of classification methods. However, there are some shortcomings. First, researchers try different models to enhance the identification rate of suicidal ideation, but ignore the application of ensemble methods. Second, few studies have focused on the effects of different feature combinations on prediction results. Meanwhile, past studies faced some common problems, such as poor quality of research data [26], homogeneity of research platforms [22], and systematic errors [24]. Therefore, this study proposes a feature combination-based ensemble method that considers feature selection to avoid overfitting problems and applies it to a real social media dataset, which fills the research gap to some extent.

## 3. Materials and Methods

### 3.1. Data Set

In this study, we chose Weibo as the data source, a Chinese social media platform similar to Twitter. The platform allows users to share and spread information instantly and interactively in the form of text, pictures, videos, and other multimedia. The number of monthly active users of Weibo increased to more than 500 million by 2022 [32], with nearly 80% of the platform being young users. Users have the option to hide personal information and share their thoughts openly on the platform, which has attracted many depressed people to share their suicide plans on the platform. The dataset consisted of 40,222 tweets, of which 2272 had suicidal ideation and 37,950 had no suicidal ideation. Depression is the important risk factor for suicide, so these postings about depressive tendencies are valuable for studying whether users have suicidal ideation. In addition, to protect user privacy, personally identifiable information (such as user ID, user nickname) was not included in the data.

In the study of Wang [33], notes for suicide rating were designed according to the Hamilton depression Scale [34] and Zimmerman’s work [35], which inspired us to establish notes standards. A post will be classified as having suicidal ideation only if it contains not only a suicide plan, but also a specific plan to commit suicide. Posts that express only depressive tendencies or habitual suicidal expressions will be classified as non-suicidal. In addition, the dataset will exclude some samples where the posting content is meaningless or the context is simple and undecipherable. Specific categories and examples are shown in Table 2.

In order to ensure the consistency of data coding, four researchers independently annotated a group of random microblogs (*n* = 500) after simple training, and tested the differences in researchers’ coding results by the intra-group correlation coefficient (ICC). At the completion of the first labeling round, the degree of agreement was (ICC = 0.658, *p* < 0.001). After discussion and analysis of the inconsistent microblogs, the annotators randomly selected 500 microblogs again for annotation, and the consistency of this coding round was (ICC = 0.885, *p* < 0.001).

### 3.2. Feature Construction

#### 3.2.1. Basic Statistical Characteristics

According to the original post information, we calculated the language and time characteristics, respectively. Language features cover the user’s preference for expressing in different categories of languages, the use of emotional vocabulary, emojis, and so on. We used the Chinese psychological analysis software ‘TextMind’ [36] to count the frequency of different word categories of posts. It is developed based on LIWC2007 and C-LIWC dictionaries, realizing a one-stop solution from automatic Chinese word segmentation to psycholinguistic analysis. LIWC [37] has been widely used in linguistic feature analysis, including 7 major psycholinguistic categories and 61 subcategories. Due to the differences between Chinese and English language styles, TextMind, facing the Chinese language environment, can better analyze users’ language preferences. Psychological studies have shown that suicide is a cumulative cause [38], and that the cumulative and repeated outbreaks of negative emotions are the triggers of suicide. In order to evaluate the intensity of expression of negative and positive emotions, several Chinese emotional dictionaries are integrated to count the occurrence of positive and negative emotional words, including the Dalian University of Technology dictionary, the HowNet dictionary, the NTUSD simplified Chinese dictionary, and the Tsinghua University Li Jun Chinese praise and derogation dictionary. Unstable and rapidly fluctuating emotional patterns are strongly associated with an individual’s suicide risk [39], and degree adverbs are often used to modify psychological verbs to reinforce or weaken emotional intensity. Similar to the statistical method of emotion words, the frequency of occurrence of adverbs of the degree of four levels was counted for each post. In addition, the occurrence of certain emojis in suicide posts can also be a red flag, such as the emojis ‘Drugs’ and ‘Knife’, which indicate specific ways in which suicide is carried out. Studies have shown that there are also time patterns for suicide, and sleep disorders can significantly increase the risk of suicide ideation and even suicide death [40]. We divided a day into 8 fixed time periods (8 categories) of 3 h each, and then categorized the time of each post.

#### 3.2.2. Risk Factors for Suicide

Vocabulary has proven successful in efforts to screen for various types of psychiatric disorders in online communities. We chose the Chinese suicide dictionary to count the frequency of suicide words in posts. It is constructed based on the content pool of posts made by Weibo users who have committed suicide, and has achieved good results in assessing the level of suicide risk of users [41]. It is worth noting that all the words in the dictionary are grouped into 13 different categories that relate to different aspects of user expression in suicide. It also mapped out risk factors strongly associated with suicide, such as vocabulary related to self-mutilation (hanging, falling, carbon); vocabulary that reflects trauma or unpleasant experience and life pressure (lovelorn, extramarital affairs, death, debt repayment); and vocabulary for talking about relatives and friends around (classmates, parents, friends). There are also words about psychiatric disorders and somatic symptoms (bipolar disorder, regurgitation, sleep), and certain discourse implying anger, hopelessness, shame and guilt (damn it, leave it, apology). Suicide triggers are often reflected in the expression of suicidal ideation, such as stress [42], mood [43,44], depression [45], and life experiences [46], have been shown to be the core factors of suicidal behavior.

#### 3.2.3. Word Embedding Clustering

Word embedding is one of the important technological breakthroughs in natural language processing. It is the representation of text data as a real number vector, and the use of the word embedding process is to map each word in the vocabulary to a real number vector on a low dimensional space in a predefined vector space [47]. Currently, many deep learning prediction models use word embedding techniques to characterize text information features. Compared with traditional text representation, word embedding captures contextual information between words. This advanced technology has also been applied to suicide risk assessment in recent years [16]. Therefore, based on the Word2vec word vector, we use the K-means algorithm to cluster keywords highly related to suicide risk. The specific steps are as follows:

(1) Using the Skip-gram model to train the word embedding model, generate and save the vector file of word embedding.
(2) Jieba word segmentation tool is used to perform data preprocessing operations such as word segmentation, part-of-speech tagging on suicide text D, reserving the part-of-speech words containing the main content of the text, and obtaining N candidate keywords, namely D = [t1, t2, …, tn].
(3) Traverse and extract the candidate keyword vector from the word embedding vector file, i.e., WV = [v1, v2, …, vm].
(4) The K-mean algorithm is used to cluster keywords. First, randomly assign words as initial centers, candidate keywords are categorized into the nearest cluster, then recalculate the cluster centers, and repeatedly assign and update the cluster centers until the cluster centers are not changing.
(5) Calculate the distance from candidate keywords in the cluster to the cluster center, and determine text keywords according to distance size.

### 3.3. Experimental Design

This study suggests that three different functional features (basic statistical characteristics, risk factors for suicide, and word embedding clustering) can complement each other and compensate for their deficiencies. The basic statistical characteristics include dictionary LIWC statistics, emotional vocabulary, emojis, etc. Although LIWC includes basic psychological features, the specific expression of suicide cannot be comprehensively covered. Therefore, we selected the features of suicide risk factors to expand the key information about suicide expression, such as the suicide tools and methods mentioned by users when expressing suicidal ideation, as well as the expression of various suicide inducements. However, the basic statistical characteristics and suicide risk factors did not consider the word order information, syntactic structure and semantic information of posts. The Word2vec method in word embedding clustering can extract key content by combining the context information of users’ posts [47], taking more account of the semantic information of posts, and getting the most important contribution features by further clustering. In other words, the features extracted by the word embedding cluster can serve as a complement to the basic statistical characteristics and suicide risk factor characteristics. These three methods, respectively, express the basic information of posts from different perspectives, focusing on the different contents of the posts.

To achieve an efficient classification of suicidal ideation posts, we constructed a classification model by fusing the three features in different combinations. The machine learning algorithms employed vary with the effect of the basic classifier for each feature. This is essentially a hybrid approach, with differences in the basic classifiers for each set of features, which further improves suicide ideation recognition performance by integrating the best models selected based on the characteristics. Therefore, there are three stages to implementing a customized ensemble learning program.

Single feature classification: Several classification algorithms are implemented to evaluate the performance of basic statistical characteristics, suicide risk factors, and word embedding clustering features. Meanwhile, it is difficult to avoid the text being vectored into a high-dimensional sparse matrix because the original information of the post is represented from three perspectives. To address this problem, we apply the feature engineering technique—extreme random forest—to mitigate dimensional disasters. The basic model mainly selects four support vector machines with different kernel functions [48], Bayesian algorithms [49], K-nearest neighbor algorithm [50], logical regression [51], decision tree [52], and extreme random forest [53]. The parameters of the first five algorithms are default parameters, while decision tree and extreme random forest selection are two ways to evaluate the importance of nodes, namely the Gini coefficient and entropy.

Multi-feature classification: In this stage, three features are combined in different ways and can be simply divided into two-dimensional combination connections (BSC + WEC, BSC + RFS, WEC + RFS) and three-dimensional combination connections (BSC + WEC + RFS). Furthermore, we again applied feature selection steps and compared the effects under feature selection, followed by combination and feature combination, followed by selection, respectively. Similarly, several algorithms are applied to select the best classification model for each feature combination. The purpose of this is because the features interact or relate with each other after transformation, and these fused correlations may affect the classification effect.

**Figure 1:**
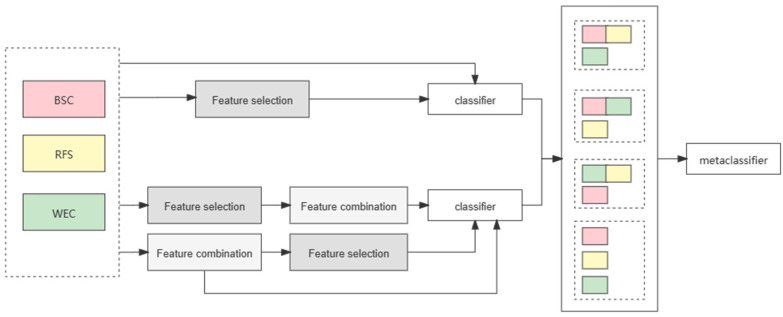
Suicide ideation detection framework. Note: BSC = basic statistical characteristics, RFS = risk factors for suicide, WEC = word embedding clustering.

Ensemble classification of feature fusion: This stage uses a combination of single and multiple features to construct a feature set, each of which covers all the original features. This ensures that the basic classifier can obtain information expressed in different ways in suicide posts in each feature set, avoiding the identification of suicidal ideation as a one-sided judgment. Similarly, some features are processed by feature dimension reduction, and the output results are obtained through five-fold cross-validation. For each feature, the best basic classifier was selected for prediction, and the prediction results will be entered as a new feature into the meta-classifier to make a further judgment. Here we select logistic regression as a meta-classifier to integrate the prediction results of the basic classifier. The specific processing framework is shown in Figure 1.

## 4. Results

### 4.1. Single Feature Classification

In order to obtain the best classifier for a single feature, each feature is input into several classification algorithms. The output of each classification model was obtained by five-fold cross-validation. The accuracy, F1-score, precision and recall value of the best models with different features are shown in Table 3. We have observed that the performance of all three types of features has been improved to varying degrees by dimensionality reduction. The performance of word embedding clustering features improved most obviously after feature selection, with the accuracy and F1-score, respectively, increasing by 1.51% and 2.50%. Among them, suicide risk factors were more powerful in differentiating suicidal ideation, with an accuracy of 76.19% and an F1-score of 72.77%.

### 4.2. Multi-Feature Classification

Table 4 shows all combination schemes of the three features, including direct combination, feature selection followed by combination, and feature combination followed by selection. Similarly, we still choose the best classifier for each model to show. First, the comparison between two-dimensional feature combinations showed that suicide risk factors and word embedding clustering (RFS and WEC) performed better than the other two combinations (BSC and RFS, BSC and WEC) in the two mixed modes of direct combination and feature selection followed by a combination. However, it is worth noting that in the pattern of dimensionality reduction after combination, the combination of basic statistical characteristics and suicide risk factors is superior to the combination of the other two types of features; that is, the model classification effect of (BSC + RFS)-fs is superior to (BSC + WEC)-fs and (RFS + WEC)-fs. Among the three-dimensional feature combinations, the best performing model was (BSC + RFS + WEC)-fs, with a prediction accuracy and F1-score of 80.15% and 78.60%.

### 4.3. Ensemble Classification of Feature Fusion

Table 5 shows the performance of our proposed model over different feature sets. When evaluating the performance of the improved models in different feature sets, we observed that the classification model performance of the feature sets (BSC + RFS)-fs and (WEC-fs) outperformed the other feature sets with 80.61% accuracy and a 79.20% F1-score, which were the highest performance results. Where all three classes of features were used (the last three rows of Table 3), most results from the improved model outperformed the performance of the single classification models, except for the features (BSC, RFS, WEC) and ((BSC-fs) + (RFS-fs), and WEC-fs).

In order to verify the validity of the proposed model, several popular ensemble learning methods (random forest, gradient boosting, XGBoost, AdaBoost, bagging and stacking) are selected for experimental comparison. Among them, random forest chooses two ways to evaluate the importance of nodes, namely, the Gini coefficient and entropy, AdaBoost and Bagging choose the default base classifier as decision tree, and XGBoost adopts a tree-based structure and linear model to run; as such, we can compare the differences brought by different hyperparameters. Each basic classifier in the stacking ensemble method is built using the best classifier tested previously. According to the experimental results in Table 6, our results are still superior to those of other ensemble learning methods. Stacking is the best model among them, with an accuracy of 79.77% and an F1-score of 77.92%.

## 5. Discussion

Early identification and intervention are necessary to prevent suicide. Predicting users’ suicidal ideation based on social media data can avoid non-real-time and subjective problems caused by traditional self-report methods. A critical review of the literature related to social media suicidal ideation detection points out that different classification models and feature inputs lead to heterogeneity in the final results, while integrated models generally outperform single models. The result is improved by the integrated model based on feature selection proposed in this paper. In our study, multiple valid features were extracted from the user’s post content, including basic statistical characteristics, suicide risk factors, and word embedding clustering features. Due to the common characteristics of text features, there are a lot of redundant variables in the original feature set. In order to avoid dimension catastrophe, the extreme random forest method is applied to dimension reduction.

First, we determine the best classifiers for single and multidimensional features. Based on the three types of single feature classification, we noticed that the prediction performance improved to various degrees after applying feature selection techniques to each feature type. Notably, no matter whether feature selection techniques were applied for feature processing, the most valuable information was found in suicide risk factors. With increasing emphasis on suicide risk factor screening, we can further use machine learning to determine which factors are most important for capturing risk [54].

When using multidimensional feature classification, three types of features are fused in different ways, and three mixed modes are tested, respectively, including direct combination, feature selection followed by combination, and feature combination followed by selection. The results prove that the combination of features is critical, and that proper blending of different functional features helps improve predictive performance. Different forms of text representation contain different contents about suicide information, and various functional carriers will make up for the information differences between them. After single feature and multidimensional feature classification, the best classifier of different features can be determined. Our proposed model can be used to integrate the best classification model of the first two stages. To verify the effectiveness of the improved model, we compared it with the existing multiple ensemble models. The results showed that our model had better performance relative to previous research models, with the accuracy and F1-score of 80.61% and 79.20%, respectively, for identifying posts with suicide ideation. The ensemble method constructed by us integrates the advantages of various features to avoid one-sidedness in recognition. Instead of passing all the feature spaces to each classifier, each group of features in our model inputs its corresponding best-performing classification model. Basic classifiers trained in different subspaces in the features space can notice different patterns in suicide data, and selecting the optimal classifier can maximize the advantages of each group of features to make more accurate predictions. This also proves that the selection of basic classification models significantly impacts the overall predictive performance [55].

## 6. Conclusions

To help public health professionals quickly identify suicidal individuals and thus better provide healthcare, this study utilizes machine learning techniques to identify posts of suicidal individuals and, to some extent, improve our prediction accuracy. Unlike general methodological studies, we further introduce our research model by presenting a critical review of the field to demonstrate the latest research advances. We also design a rigorous experimental procedure to test our model with a large amount of data objectively available on Weibo platforms. Our results show that the ensemble model based on feature combination extracts more feature information and has a unique advantage in detecting suicidal ideation. Our study, to some extent, bridges the gaps and provides new insights from existing studies.

### 6.1. Theoretical Contribution

The results of this study reveal several critical theoretical contributions. First, this study contributes to the literature on public health and safety by innovatively proposing an ensembled approach based on feature combinations, which enriches the idea of model construction based on the full reference of previous research results. Second, this study highlights the current status and shortcomings of existing research in the literature through a critical review of research in the field, and presents it in a tabular format for researchers to draw on and reference.

### 6.2. Practical Implications

This study also provides practical implications in the following ways. First, the use of machine learning techniques to analyze social media content can be beneficial in helping physicians identify and intervene in a timely manner with potentially suicidal populations [5]. Although the method proposed in this paper only improves the performance of existing suicide prediction models, continuous improvement has a positive effect on the overall suicide rate reduction. Second, machine learning methods based on large-scale Internet data can also provide useful information for suicide prevention efforts, avoiding to some extent the subjectivity of questionnaires, which is important for the facilitation of clinical medical practice.

### 6.3. Limitations and Future Work

This study also has some limitations. Considering the privacy settings of Weibo, we cannot obtain users’ age, gender, location, etc. Users in Weibo communities are mostly young users. Thus, our data have some bias, while the occurrence of suicidal behavior and the relationship between age and gender have also been reported [56]. In the ensemble model, more diversified basic classification models can be considered, such as relevant deep learning algorithms. In addition, we can also develop a real-time suicide monitoring system as an effective interference point between high-risk users and mental health services.

## Figures and Tables

**Table 1:** A critical review of different suicide ideation detection studies on social media.

References	Features Extracted	Methodology Used	Social Media	Performance and Drawback(s)
[18]	Vocabulary	Naive Bayes	Twitter	Performance: Accuracy is 0.6315 in Leave One Out validation (LOO)and 0.6327 in 10-CV. Drawback: The functional features are single, and the vocabulary size limits the classification effect.
[21]	Word bags, Polarity dictionary, LSA topic model, Named entities	LIBSVM	Dutch-language forum	Performance: F1 is 0.93 for relevant messages, 0.70 for severe messages. Drawback: There is likely to be noise in user-generated content, which may influence the conclusions of the study.
[22]	Simplified Chinese-Linguistic Inquiry and Word count	Logistic regression, SVM	Weibo	Performance: The overall classification performance was not satisfactory and could only be classified among those with high probability of suicide (AUC = 0.61, *p* = 0.04) and severe anxiety (AUC = 0.75, *p* < 0.001). Drawback: The research is based on a single platform, and the generalizability of the research findings needs to be verified.
[23]	TFIDF, Word frequencies, Information retrieval	SVM	Twitter	Performance: Accuracy is 0.76 when sets A and B were combined. Drawback: Need to improve models to capture contextual information.
[24]	Demographic features, Emotion labels,	Logistic regression	Twitter	Performance: F1 is 0.53. Drawback: Those who attempted suicide in the study sample survived, and there may be systematic errors in the data.
[25]	N-gram, Word vectors, Document vectors	Random Forest, SVM, CEM, deep learning, ensemble models	Microblogging and movie reviews domain	Performance: The F1-scores are 0.7302, 0.6379, 0.7532, 0.7181, 0.8120 and 77.41 in the six datasets, respectively. Drawback: The performance effect of the model is pursued, but the interpretability of the model is ignored.
[26]	TFIDF, Linguistic inquiry and Word count, and Sentiment analysis	Logistic regression, random forest, and SVM	Reddit	Performance: Logistic regression (F1: 0.78–0.92, Accuracy: 0.76–0.92); random forest (F1: 0.75–0.92, Accuracy: 0.71–0.89); SVM (F1: 0.73–0.92, Accuracy: 0.76–0.92). Drawback: Research data are limited to English text.

**Table 2:** Examples of suicidal ideation and non-suicidal ideation posts.

Category	Example
Suicidal ideation	Anybody here? What if I swallowed 6 Escitalopram Oxalate tablets and 2 Zopiclone tablets?
	I hide in the wardrobe with a knife in my hand that can cut off the carotid artery at any time. I don’t want to work with you or see you. Don’t talk to me or save me.
Non-suicidal ideation	Today, my throat hurts more and more. I’m afraid my body is getting worse and worse. If I die, no one will care.
	I still find it extremely painful to be alive.

**Table 3:** Optimal model performance of a single feature.

Feature	Accuracy	F1-Score	Precision	Recall	Optimum Classifier
BSC	74.49%	72.41%	77.72%	67.78%	ET-g
BSC-fs	74.65%	72.34%	78.28%	67.24%	ET-e
RFS	75.86%	72.47%	83.29%	64.14%	SVM-l
RFS-fs	76.19%	72.77%	84.04%	64.17%	SVM-l
WEC	69.81%	62.98%	80.99%	51.52%	Log-l2
WEC-fs	71.32%	65.48%	81.89%	54.55%	NB

-fs = feature selection.

**Table 4:** Optimal model performance of multidimensional features.

Features	Feature Combination	Accuracy	F1-Score	Precision	Recall	Optimum Classifier
BSC RFS	BSC + RFS	78.06%	76.07%	82.76%	70.38%	Log-l2
	(BSC-fs) + (RFS-fs)	78.56%	76.84%	82.44%	71.95%	ET-g
	(BSC + RFS)-fs	78.87%	76.56%	85.10%	69.58%	SVM-l
BSC WEC	BSC + WEC	77.24%	74.84%	82.60%	68.41%	Log-l1
	(BSC-fs) + (WEC-fs)	77.60%	75.28%	82.97%	68.89%	Log-l1
	(BSC + WEC)-fs	77.70%	75.61%	81.93%	70.20%	ET-e
RFS WEC	RFS + WEC	78.17%	76.05%	83.57%	69.77%	SVM-l
	(RFS-fs) + (WEC-fs)	78.89%	76.94%	84.05%	70.94%	SVM-l
	(RFS + WEC)-fs	78.83%	76.90%	83.78%	71.06%	SVM-l
BSC RFS WEC	BSC + RFS + WEC	79.20%	77.03%	84.71%	70.63%	ET-g
	(BSC-fs) + (RFS-fs) + (WEC-fs)	79.86%	78.32%	84.05%	73.32%	SVM-l
	(BSC + RFS + WEC)-fs	80.15%	78.60%	84.44%	73.52%	SVM-l

-fs = feature selection.

**Table 5:** Suggested model performance on different feature sets.

Feature Set		Accuracy	F1-Score	Precision	Recall
	BSC + RFS	79.40%	77.70%	83.70%	72.50%
(BSC + RFS) + WEC	WEC				
	(BSC-fs) + (RFS-fs)	79.12%	77.19%	83.97%	71.42%
	WEC-fs				
	(BSC + RFS)-fs	80.61%	79.20%	84.58%	74.46%
	WEC-fs				
	BSC + WEC	79.80%	78.27%	83.64%	73.55%
(BSC + WEC) + RFS	RFS				
	(BSC-fs) + (WEC-fs)	80.11%	78.65%	84.01%	73.93%
	RFS-fs				
	(BSC + WEC)-fs	79.93%	77.85%	85.68%	71.33%
	RFS-fs				
	RFS + WEC	79.55%	77.43%	85.15%	70.99%
(RFS + WEC)+ BSC	BSC				
	(RFS-fs) + (WEC-fs)	79.78%	77.81%	85.12%	71.66%
	BSC-fs				
	(RFS + WEC)-fs	79.67%	77.64%	84.58%	71.75%
	BSC-fs				
BSC + RFS + WEC	BSC, RFS, WEC	78.92%	76.54%	85.06%	69.57%
	BSC-fs, RFS-fs, WEC-fs	80.15%	78.17%	85.73%	71.84%

-fs = feature selection.

**Table 6:** Performance results of multiple ensemble methods.

Model	Parameter	Accuracy	F1-Score	Precision	Recall
Random forest	criterion = ‘entropy’	76.78%	74.03%	82.23%	67.32%
	criterion = ‘gini’	76.43%	73.63%	81.85%	66.91%
XGBoost	booster = ‘gbtree’	77.24%	76.01%	79.60%	72.73%
	booster = ‘gblinear’	73.26%	71.65%	75.83%	67.91%
AdaBoost	base_estimator = tree	77.82%	76.78%	79.81%	73.97%
Bagging	base_estimator = tree	77.07%	75.01%	80.96%	69.87%
Gradient Boosting	/	78.76%	77.14%	82.38%	72.53%
Stacking	base_estimator = SVM-l,Log1,Log2,NB, ET-g,ET-e	79.77%	77.92%	84.76%	72.10%
Suggested model	/	80.61%	79.20%	84.58%	74.46%

## Data Availability

Not applicable.
